# Sequencing, Expression, and Functional Analyses of Four Genes Related to Fatty Acid Biosynthesis During the Diapause Process in the Female Ladybird, *Coccinella septempunctata* L.

**DOI:** 10.3389/fphys.2021.706032

**Published:** 2021-08-19

**Authors:** Mei Xiang, Hong-Zhi Zhang, Xiao-Yu Jing, Meng-Qing Wang, Jian-Jun Mao, Yu-Yan Li, Lian-Sheng Zang, Li-Sheng Zhang

**Affiliations:** ^1^Institute of Plant Protection, Chinese Academy of Agricultural Sciences, Beijing, China; ^2^Institute of Biological Control, Jilin Agricultural University, Changchun, China; ^3^Department of Entomology and BIO5 Institute, University of Arizona, Tucson, AZ, United States

**Keywords:** *Coccinella septempunctata*, diapause, cloning, expression, RNAi

## Abstract

The ladybird *Coccinella septempunctata* L., a predatory insect, serves as an excellent biological control agent against common agricultural pests. It undergoes a diapause phenomenon, during which a large amount of fat accumulates in the abdomen. A comprehensive analysis of this lipid accumulation can reveal the molecular mechanisms underlying diapause regulation, which can be exploited to improve the shipping and transport of the insect for agricultural applications. In this study, we compared the transcriptome of *C. septempunctata* during non-diapause, diapause, and post-diapause and screened four key genes related to lipid metabolism. The cDNA of these four relevant enzymes, acetyl-CoA carboxylase (ACC), long-chain fatty acid-CoA ligase (ACSL), elongase of very-long-chain fatty acids (ELO), and very-long-chain 3-oxoacyl-CoA reductase (KAR), were cloned using reverse transcription-polymerase chain reaction and rapid amplification of cDNA ends. Their expression profiles were analyzed during the preparation and maintenance phases of diapause and the post-diapause phase. The functions of these four key enzymes in diapause were further verified using RNA interference. All four genes were most closely related to the homeotic gene from *Tribolium castaneum*. The expression profiles of these four genes were significantly affected under diapause-inducing conditions; their expression level was the highest in the diapause preparation phase, and it gradually decreased with the diapause induction time. RNA interference showed that the target genes play important roles in fat storage during early diapause, and the decrease in their expression leads to a decrease in lipid content in *C. septempunctata*. These results indicate an important role of *ACC*, *ACSL*, *ELO*, and *KAR* in lipid accumulation. Our findings could help elucidate the production and accumulation of lipids by insects during the preparation for diapause and improve biological control.

## Introduction

The widely distributed seven-spotted ladybird *Coccinella septempunctata* L., a typical natural predator of agricultural pests, preys on various types of aphids, commonly found in fruit trees and crops. *Coccinella septempunctata* is distributed across various regions (Hodek and Michaud, 2008; Simelane et al., 2008). It has a characteristic diapause period during which the adult diapause elytra are light in color and remain yellow for a long period. Ovarian development is halted and a large amount of fat accumulates in the abdomen (Wang et al., 2013). This physiological change can provide energy required by the insect to withstand the diapause period, thus ensuring its survival in a hostile environment (Ren et al., 2016). Further understanding of the mechanisms regulating diapause can help improve long-distance shipping and long-term storage of this insect (Ren et al., 2016), thereby promoting its important role as an agricultural commodity in biological control (Turnock et al., 2003).

Diapause is a physiological and ecological strategy acquired during the long-term evolution of insects to enable their adaptation to adverse environments (Tougeron, 2019). Diapause is a genetically controlled phenomenon in which insect development stagnates under the stimulation of a specific environmental clue and is reinitiated under another specific stimulus (Danilevsky et al., 1970; Denlinger, 2002). Diapause is essential to species survival as it induces a rhythm in the life cycle that is in sync with the rhythm of the environment and ensures that an active phase of the life cycle occurs when food is most abundant (Andrewartha, 2010). In addition to gaining a better understanding of insect survival mechanisms, in-depth investigation of the process of diapause can also provide insights into aging, obesity, and disease transmission; help predict pest infestations; increase the shelf life and effectiveness of biological control agents; and provide new guidance for medical research (Denlinger, 2008). In particular, research into the regulation mechanism of *C. septempunctata* diapause can provide strategies for the development and utilization of this economic insect as a theoretical basis for the biological control of pests in agriculture and forestry, while further extending the shelf life of products to improve their utilization rate (Tauber and Tauber, 1976; Van Lenteren, 2012).

Lipid accumulation is critical to insect diapause regulation (Hahn and Denlinger, 2011). Thus, higher lipid levels during diapause have been reported in many insects including *C. septempunctata* (Amiri and Bandani, 2013; Wang et al., 2013; Güney et al., 2021). However, the key enzymes involved in this regulation and their specific roles in fatty acid metabolism remain unclear. The insect fatty body is the power source to maintain the energy balance *in vivo* and serves as an essential reservoir to store fat during the long period of fasting throughout the process of insect metamorphosis (Li et al., 2019). After being absorbed by the insect, most nutrients are stored *in vivo* in the form of triglycerides (Sieber and Thummel, 2009). In most insects, the majority of total fatty acids are associated with the synthesis of triglycerides, which play an important role in maintenance and survival (Toprak et al., 2020). Several key genes participate in the fatty acid biosynthesis and elongation pathways (Stanley-Samuelson et al., 1988). We earlier performed transcriptome sequencing of insect in three development stages, namely, non-diapause, diapause, and diapause termination. Differentially expressed genes were screened, which revealed that differentially expressed genes were primarily involved in the fatty acid synthesis pathway (Qi et al., 2015). According to the *de novo* transcriptome analysis of *C. septempunctata* [available at the National Center for Biotechnology Research (NCBI) Gene Expression Omnibus under accession number GSE75645], we screened four genes associated with fatty acid biosynthesis that are significantly upregulated during diapause, namely, acetyl-CoA carboxylase (*ACC*), long-chain fatty acid-CoA ligase (*ACSL*), elongase of very-long-chain fatty acids (*ELO*), and 3-ketoacyl CoA reductase (*KAR*). ACC and ACSL play crucial roles in fatty acid metabolism. ACC is the rate-limiting enzyme in the *de novo* synthesis of fatty acids, which catalyzes the production of acetyl-CoA, eventually catalyzing the synthesis of palmitic acid (C16:0) and distinct elongases in the pathway of very-long-chain fatty acyl-CoA synthesis (>C22:0) (Barber et al., 2005; Tong, 2005). ACC has been used in drug design for obesity and diabetes, as well as herbicide formulations, thereby becoming a target gene for some crops; Moreover, ACC is a target for lipid synthesis inhibitor type insecticides/acaricides (Herbert et al., 1997; Abu-Elheiga et al., 2003; Lümmen et al., 2014). Insect ACC is a multidomain enzyme encoded by a single gene, affecting lipid accumulation, reproductive capacity, and insects’ epidermal function (Baker and Thummel, 2007). Long-chain fatty acid-CoA ligase activates free long-chain fatty acids to form acyl-CoA in the fatty acid biosynthesis pathway. Acyl-CoA is a key intermediate of anabolism and catabolism (Mashek et al., 2007; Dai et al., 2015), whereas ACSL is involved in fatty acid transport, energy balance, post-translational modification of proteins, cell signaling, and cell wall synthesis (Watkins, 1997). The activated form of acyl-CoA participates in the extension cycle of the fatty acid elongation pathway. After four sequential reactions of condensation, reduction, dehydration, and final reduction, long-chain fatty acids are generated (Moon and Horton, 2003). Elongase of very-long-chain fatty acids and KAR are the key enzymes in this reaction. The pathway is catalyzed by ELO, and malonyl-CoA provides two carbon atoms, which extend fatty acid carbon chains to generate 3-ketoacyl-CoA. 3-Ketoacyl CoA reductase then catalyzes the reduction reaction to form 3-hydroxyacyl-CoA (Leonard et al., 2004). Research on ELO in insects has mainly focused on *Drosophila melanogaster* to elucidate its role in reproductive ability, pheromone biosynthesis, and epidermal function (Falcón et al., 2014; Ng et al., 2015). 3-Ketoacyl CoA reductase is a critical factor in the very-long-chain fatty acid prolongation enzyme complex, widely studied in plants such as *Brassica napus* and cotton fibers, but its role has rarely been reported in insects (Puyaubert et al., 2005; Pang et al., 2010).

In this study, we cloned these four genes related to fatty acid biosynthesis in *C. septempunctata* and used reverse transcription-quantitative polymerase chain reaction (RT-qPCR) to detect the gene expression levels during non-diapause and diapause induction. RNA interference (RNAi) technology was further used for genetic silencing to confirm the functions of these genes in diapause process. These results can provide a theoretical basis for a more in-depth understanding of diapause molecular regulation and provide a new direction for the commercial application of *C. septempunctata*.

## Materials and Methods

### Insect Rearing and Sample Collection

*Coccinella septempunctata* were captured in wheat fields in the Chinese Academy of Agricultural Sciences and reared on fresh *Aphis glycines* Matsumura daily (Wang et al., 2013). Female adults exhibiting normal development were maintained under suitable conditions, with a photoperiod of 16-h light:8-h dark, temperature of 24 ± 1°C, and humidity of 70 ± 10%. To induce diapause, the newly emerged adults were shifted to diapause conditions with a reduced day length of 14-h light:10-h dark, low temperature of 18 ± 1°C, and normal humidity of 70 ± 10%. Adults raised under normal developmental conditions were used as non-diapause group until the first oviposition. The newly emerged adults obtained under normal developmental conditions were separated into male-female pairs and transferred to the diapause induction conditions for 10, 20, 30, and 60 days; 10 and 20 days of diapause induction were used as the preparation period, 30 days of diapause induction was used as the diapause period, and 60 days of diapause induction was used as the late stage of diapause. Adults in diapause were moved to normal developmental conditions until their first oviposition, as the post-diapause group. Diapause assessment was based on the method of Wang et al. (2013). An insect was assessed to be in a diapause state when the normal prooviposition was prolonged by two times and the development of immature eggs and oocyte formation almost stopped in the ovary, as observed by dissection. For the collection of *C. septempunctata* from each treatment group, we selected newly emerged female adults, non-diapause females, non-oviposition females adults at 10, 20, 30, 60 days after diapause induction, and the post-diapause female adults (*n* = 1–330), which were then washed with distilled water, dried on filter paper, and frozen with liquid nitrogen until analysis.

### Cloning of Full-Length cDNA and Sequencing

#### Cloning of Full-Length cDNA

The total RNA was extracted from individual *C. septempunctata* 60 days after diapause induction using RNAiso Plus (TaKaRa, Dalian, China) in accordance with the manufacturer’s instructions. The RNA purity and concentration were determined by 1.5% agarose gel electrophoresis and spectrophotometry using P-class NanoPhotometer (Implen, München, Germany), respectively. The first strand of cDNA used for internal sequence amplification was synthesized using the Glodenstar RT6 cDNA Synthesis Kit (TSINGKE, Beijing, China). The obtained cDNA was stored at –20°C. Four fragments encoding putative *ACC* (contig_10367), *ACSL* (contig_10754), *ELO* (contig_4307), and *KAR* (contig_18276) were identified based on the transcriptome data. Specific primers were designed (Table 1) and synthesized by TSINGKE Biological Technology Company (Beijing, China). PCR was then performed with I-5 2 × High Fidelity Master Mix (TSINGKE). The PCR products were separated on a 1.5% agarose gel and purified using NucleoSpin Gel and PCR Clean-up (Macherey-Nagel, Duren, Germany). The target genes were then subcloned into the pClone007 Blunt Simple Vector Kit (TSINGKE) and sequenced by Sangon Biotech (Shanghai, China). Full-length cDNA was obtained by 5′ and 3′ rapid amplification of cDNA ends (RACE) using the SMARTer RACE 5′/3′ Kit (Clontech, Mountain View, CA, United States) according to the manufacturer’s instructions. The final amplification products were purified as described above. The isolated fragments were cloned and transformed using the In-Fusion HD Cloning Kit (Clontech) and then sequenced.

**TABLE 1:** Primers used for gene cloning, qPCR and dsRNA synthesis.

**Primer name**	**Primer sequence (5′-3′)**	**Primer name**	**Primer sequence (5′-3′)**
ACC-f1	ACAACGGTATAGCAGCAGT	RACE-ACCf1	GTTTCCAGGCCCTCAACAAGGTGCT
ACC-r1	AGCCTGATGTCCTTGATGTAATG	RACE-ACCf2	AGACCGGTTTACGCCTCCAATCATCA
ACC-f2	CGACAAGTGCAGACGGAGAT	RACE-ACCr1	ATGAGTTCCACGTTGGCGTAGTTGTTGT
ACC-r2	GTAGCGGTCGACTTCCTCCTT	RACE-ACCr2	CTCCGGGCACGGGTACGTAATGAT
ACC-f3	GACAACACCATAAGTACATCGTTCAA	RACE-ACSLf1	TTGAGTTCGGCTCGATAGCAATCTAGTTTC
ACC-r3	TCGAATGGTCGGAACGGTT	RACE-ACSLf2	AACCTCCAAATCCATTTGTTCCAGCAAT
ACC-f4	GACGTCGAGAGGCAGTTCCAGC	RACE-ACSLr1	TATTCGATTCTTTTGCGCCTCTTCTGA
ACC-r4	TCTGTTCGATCAGCCTGGTCT	RACE-ACSLr2	GAAGGATTCGTACAGCGTCCTGGCAT
ACSL-f1	ATACAAGCCCAAGAGCTCAGACT	RACE-ELOf1	TCGAACCTAATCAACAACATAGTACACGTTTTG
ACSL-r1	GTGTGATCGCCCTGTATGGA	RACE-ELOf2	ACATGGTTTCTGCAATGGGTCCTGA
ACSL-f2	TGCCTGTTTCAGTAAGATTATGCT	RACE-ELOr1	TGTTGTTCATCCTGGAGATTTCCATCCT
ACSL-r2	CTGAATTGTTAATTTGAAGTGCAAGT	RACE-ELOr2	TCACAAACTCCGAATCCATGGCGA
ELO-f	GATAGTAGGGTCGGTCGTT	RACE-KARf1	CCTCAACAGCGGCTCTGATACCCAA
ELO-r	TAGTCTTCAGGCTCTTGTCT	RACE-KARf2	AAGCCGCGGTTGCCAAGTTCAG
KAR-f	AGGAAGCACAGACGGTATAGGAG	RACE-KARr1	TGTCCTTTGTGAAATCCGCAGCGA
KAR-r	CCATCCAGGTGGACTTCTTGA	RACE-KARr2	GATGGTCTTGACTTCCACCTTGTACTTCTCT
qACC-f	TCACGTTCCGAGCCAGAGAC	dsACC-f	TTCAGTCACCCCCAGTCC
qACC-r	TGGTCAGGGCTTCCAGGTTG	dsACC-r	ATTTCCAAGCCACCATAC
qACSL-f	CCACGAAAACCAGGGCGAAAA	dsACSL-f	CCGTGAGTGCTGTCCTTA
qACSL-r	GTCTGTTTTGGCACCTCTCGC	dsACSL-r	GACCTCTCCCTGGTTGTT
qELO-f	TCCCGCTCGATAATCCTGCT	dsELO-f	TGGGTGGTTCTGGGATTA
qELO-r	TGGGTCGCTTATCCTGTCCA	dsELO-r	AACTGGGCTGAGTGGATG
qKAR-f	TATCTACCGCACACCCTGATGAAC	dsKAR-f	GTAGCCCAAGAAATCACA
qKAR-r	CATCATGACTCAACTGGACTTCGC	dsKAR-r	TAACCTGGACACACGCAC
Actin-f	GATTCGCCATCCAGGACATCTC	dsGFP-f	CACAAGTTCAGCGTGTCCG
Actin-r	TCCTTGCTCAGCTTGTTGTAGTC	dsGFP-r	AGTTCACCTTGATGCCGTTC

#### Sequence Analysis

Fragment assembly of the cDNA nucleotide sequence was performed using DNAMAN software. The open reading frame (ORF) and amino acid sequences were deduced using the ORF Finder program.^1^ The physicochemical properties of the proteins were computed using the online software ExPASy.^2^ TMHMM server v. 2.0^3^ was used to predict the across-membrane structure. Analysis of the conserved domains was performed using the CD Search program.^4^ Phylogenetic analyses were performed by comparing the amino acid sequences with those in NCBI using the BLAST program.^5^ Neighbor-joining phylogenetic trees were constructed using MEGA 5 software for homologous sequences obtained from the search.

### Comparative Gene Expression Profiles With qPCR

To evaluate the changes in the gene expression profiles during the diapause process, the total RNA was extracted from the insect samples of the seven treatment groups and was reverse-transcribed into cDNA using the PrimeScript Reagent Kit with gDNA Eraser (Perfect Real Time, TaKaRa, Dalian, China). qPCR was then carried out on a LightCycler 96 system (Roche, Basel, Switzerland) using the SYBR Premix Ex Taq II Kit (Tli RNaseH Plus, TaKaRa). Each 20 μL of PCR mixture contained 10 μL of SYBR green, 6.4 μL of ddH_2_O, 2 μL of DNA, and 0.8 μL of each primer. The program was as follows: initial preincubation at 95°C for 30 s, followed by 40 cycles at 95°C for 5 s and 60°C for 20 s, and a final melting curve step at 95°C for 10 s, 65°C for 60 s, and 97°C for 1 s. The standard reference gene actin was used as an endogenous control to normalize target gene expression levels (Yang et al., 2016). The sequences of the primers used for RT-qPCR are listed in Table 1. Each experiment consisted of three independent biological replicates with three technical duplicates.

### RNAi Assay

Specific primers were designed to target each gene, and T7 promoter sequences (GCGTAATACGACTCACTATAGGG) were added to the 5′ ends of the upstream and downstream primers for *in vitro* transcription and synthesis of double-stranded RNA (dsRNA) to amplify the cDNA fragments. Green fluorescent protein (GFP) was used as a negative control for evaluating the non-specific effects of dsRNA. All primers used are listed in Table 1. The PCR product of the target gene was excised, purified, and used for *in vitro* transcription. Subsequently, dsRNA for the target gene was synthesized using the MEGAscript T7 High Yield Transcription Kit (Invitrogen, Carlsbad, CA, United States) according to the manufacturer’s protocol. The dsRNAs were quantified by spectrophotometry, and their integrity was checked on 1.5% agarose gels. Purified dsRNA was diluted and packaged according to specific requirements and stored at −80°C.

Female adults of *C. septempunctata* of similar sizes were randomly selected and reared for 2 days (3 days after eclosion) under diapause-induced conditions. The internode membrane of the second and third abdominal segments of each female adult was injected with 1 μL of dsRNA solution for each target gene, and the control groups were injected with an equivalent amount of dsRNA-GFP solution. The injection concentration was 2 μg/μL. After injection, the insects were reared in pairs under diapause-induced conditions. The whole insects were collected at 3, 5, 7, 9, and 11 days after injection and stored at −80°C for subsequent RNA extraction. The specific primers used are listed in Table 1. RT-qPCR was then performed as described above to determine the mRNA expression levels of target genes after gene interference to confirm effective gene expression knockdown.

To investigate the effects of these four target genes on lipid accumulation during diapause induction, female adults of *C. septempunctata* were randomly collected at 3, 7, and 11 days after injection, washed with sterile water, dried on filter paper, and weighed; the total lipid content was determined for each period. Total lipids were extracted according to the methods of Folch et al. (1957) and Zhou et al. (2004), and the absorption was measured by vanillin-concentrated sulfuric acid colorimetry (Van Handel, 1985). There was 1 replicate established per female adult, 10 biological replicates, 10 female adults per treatment, and 3 technical replicates. Seven days after injection, the insects were dissected under a stereomicroscope to observe the development of abdominal fat bodies.

### Statistical Analysis

Data are presented as mean ± standard error by one-way analysis of variance (ANOVA). The results were compared among groups using GraphPad Prism 7.0 (GraphPad Software, San Diego, CA, United States). Gene expression levels were calculated using the 2^–ΔΔ*CT*^ method described by Livak and Schmittgen (2002), in newly emerged insects and in insects 3 days after dsGFP injection, and were used to calculate the relative expression. When a significant effect was found (*P* < 0.05), Tukey’s *post-hoc* test was performed for multiple range comparisons.

## Results

### Characterization of *C. septempunctata ACC*, *ACSL*, *ELO*, and *KAR*

The four genes related to fatty acid biosynthesis identified in *C. septempunctata* were designated as *CsACC, CsACSL, CsELO*, and *CsKAR*. The full-length sequences of *CsACC*, *CsACSL*, *CsELO*, and *CsKAR* have been deposited at GenBank^6^ under accession numbers MT012819, MT995743, MT995744, and MT995745, respectively. Sequence analysis showed that the cDNA sequence of *CsACC* is 7,217 bp long, including an ORF of 6,792 bp (position 93–6,884 bp), and encodes a protein of 2,263 amino acids. An acetyl-CoA carboxylase (ACC) domain exists at amino acids 731–1,474 of the protein, which has the typical structural characteristics of ACC. The enzyme is involved in the synthesis of long-chain fatty acids and catalyzes the rate-limiting step in this process. The cDNA sequence of *CsACSL* is 3,890 bp long, including an ORF of 2,046 bp (position 79–2,124 bp), and encodes 681 amino acids. Transmembrane structure prediction showed that the protein contains one transmembrane helical structure. The domain analysis indicated that the protein contains a long-chain fatty acid-CoA synthetase domain at amino acid residues 105–678, which catalyzes the formation of fatty acyl-CoA and activates fatty acids of chain lengths 12–20. The *CsELO* cDNA comprises 2,773 nucleotides, including an ORF of 139–1,047 bp, and encodes a protein of 302 amino acids. This protein contains six transmembrane helical structures, located between amino acids at residues 56–78, 91–113, 191–213, 226–248, and 258–280. It is predicted to be an integral membrane protein. A conserved ELO superfamily domain was identified at amino acids 54–287. Members of this family are involved in long-chain fatty acid elongation systems that produce the 26-carbon precursors for ceramide and sphingolipid synthesis. The length of the cDNA sequence of *CsKAR* is 1,091 bp, including an ORF of 945 bp (position 66–1,010 bp), encoding a protein of 314 amino acids. The protein analysis identified a 17-beta-hydroxysteroid dehydrogenase domain belonging to the SDR superfamily (residues 48–280), which catalyzes the reduction of 3-ketoyl-coenzyme A intermediates formed during each cycle of fatty acid elongation.

**FIGURE 1:**
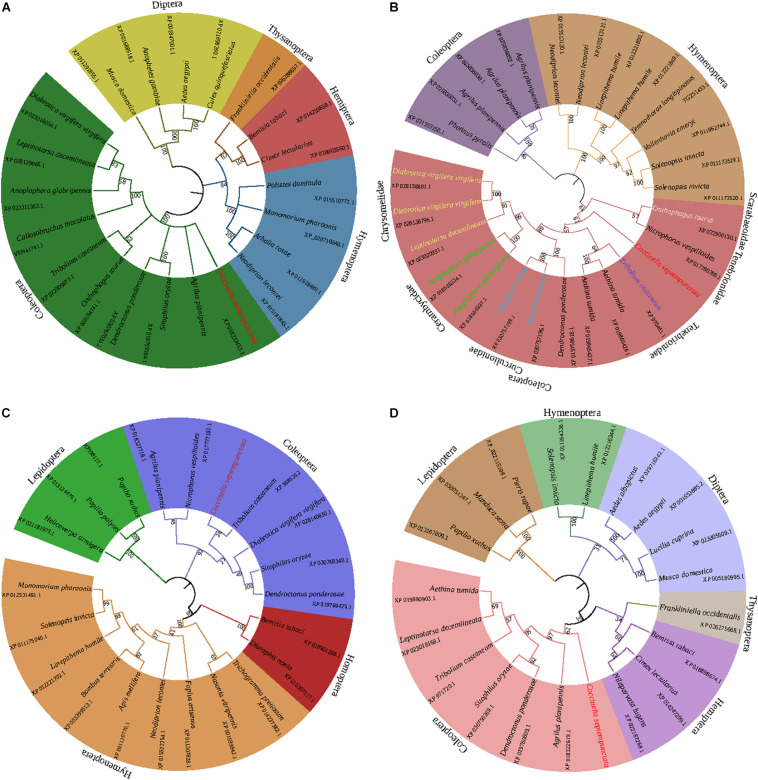
Phylogenetic trees of *CsACC* **(A)**, *CsACSL* **(B)**, *CsELO* **(C)**, and *CsKAR* **(D)**. The phylogenetic trees were constructed by the neighbor-joining method and supported by bootstrap analysis with 1,000 replications. The numbers above the branches represent the bootstrap values.

The online software ExPASy was used to predict the physicochemical properties of the CsACC, CsACSL, CsELO, and CsKAR proteins (Table 2). The neighbor-joining phylogenetic tree (Figure 1) showed that the homology among homeotic genes was consistent with the evolutionary relationships among species, and the four target genes were all most closely related to the homeotic gene from *Tribolium castaneum.*

**TABLE 2:** Physicochemical properties of CsACC, CsACSL, CsELO, and CsKAR.

**Name**	**Fragment size (bp)**	**Theoretical molecular weight (kD)**	**Theoretical pI value**	**Number of negatively charged amino acid residues (Asp + Glu)**	**Number of positively charged amino acid residues (Arg + Lys)**	**Instability index**	**Aliphatic index**	**Grand average of hydropathicity (GRAVY)**
CsACC	7,217	255.90	5.90	299	261	41.67	90.31	–0.300
CsACSL	3,890	75.77	7.88	72	75	32.03	85.99	–0.106
CsELO	2,773	35.99	8.93	24	30	33.77	94.57	0.064
CsKAR	1,091	34.60	9.04	29	39	32.80	99.97	0.159

### Expression Profiles of *CsACC*, *CsACSL*, *CsELO*, and *CsKAR* During the Diapause Process

**FIGURE 2:**
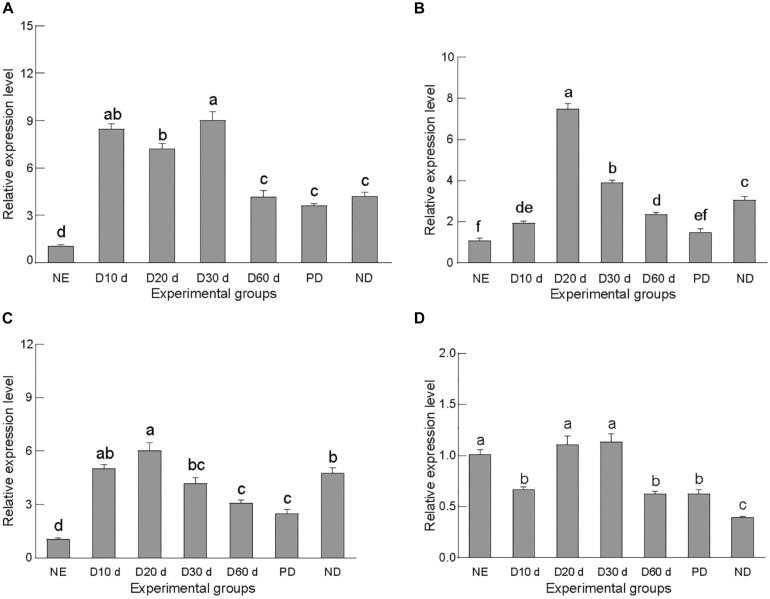
Relative gene expression of *CsACC* **(A)**, *CsACSL* **(B)**, *CsELO* **(C)**, and *CsKAR* **(D)**. NE, newly emerged adults; D10d, delayed induction for 10 days; D20d, delayed induction for 20 days; D30d, delayed induction for 30 days; D60d, delayed induction for 60 days; PD, post-diapause; ND, normal development. Data are presented as mean ± SE. Significance analyses were performed using Tukey’s *post-hoc* test. Different letters indicate a significant difference between stages (*P* < 0.05).

The four target genes were differentially expressed in *C. septempunctata* in different life stages—in newly emerged adults; in non-diapause; female adults at 10, 20, 30, and 60 days after diapause induction; and post-diapause (Figure 2). Specifically, gene expression levels were significantly higher in the early stage of diapause induction than in the non-diapause stage, consistent with the transcriptome analysis data of *C. septempunctata* (Qi et al., 2015). In the diapause induction stage, the expression of the genes increased proportionally with the induction time and peaked during the early diapause (days 20–30), at levels significantly higher than those detected in the late diapause and post-diapause. Subsequently, their expression gradually decreased with the diapause induction time, with no significant difference between the late diapause and post-diapause phases. These results suggested that all four target genes might play an important role in diapause preparation or early diapause.

### Effect of Silencing *CsACC*, *CsACSL*, *CsELO*, and *CsKAR* Expression

#### Confirmation of RNAi Efficacy

**FIGURE 3:**
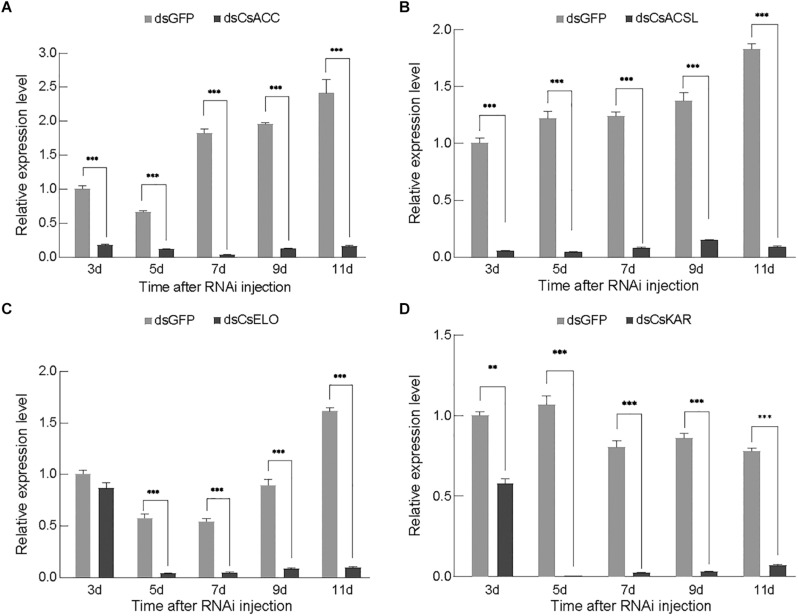
Relative gene expression of *CsACC* **(A)**, *CsACSL* **(B)**, *CsELO* **(C)**, and *CsKAR* **(D)** after dsRNA injection. Data are presented as mean ± SE. Significance analyses were performed using Tukey’s *post-hoc* test (mean ± SE; ^∗∗^*P* < 0.05; ^∗∗∗^*P* < 0.01). Different letters indicate a significant difference between stages (*P* < 0.05). **Indicated significant difference between experimental group and control group (*P* < 0.05). ***Indicated that the difference between experimental group and control group was extremely significant (*P* < 0.01).

Three days after injection, the qPCR analysis confirmed that the dsRNA effectively decreased the transcription levels of the corresponding genes compared with the dsGFP treatment in RNAi-treated females (Figure 3). The expression of *CsACSL* and *CsACC* significantly reduced 3 days after dsRNA injection (*p* < 0.0001), and they were barely expressed on days 5 and 7, with only 3.65 and 1.97% of the levels in the dsGFP control group (*p* < 0.0001), respectively. The expression of these genes subsequently increased but remained significantly lower than that in the control group (Figures 3A,B). The expression of *CsELO* and *CsKAR* was lower than that in the control group on day 3 after dsRNA injection and reached the lowest level on day 5, only 6.84 and 0.62% of the level in the control group (*p* < 0.0001), respectively. The expression of these genes then followed an upward trend (Figures 3C,D). Overall, each gene expression was significantly downregulated at different periods and then upregulated at different periods, which might have been caused by the different durations of gene silencing efficiency.

#### Impact of Gene Silencing on Lipid Accumulation in *C. septempunctata*

**FIGURE 4:**
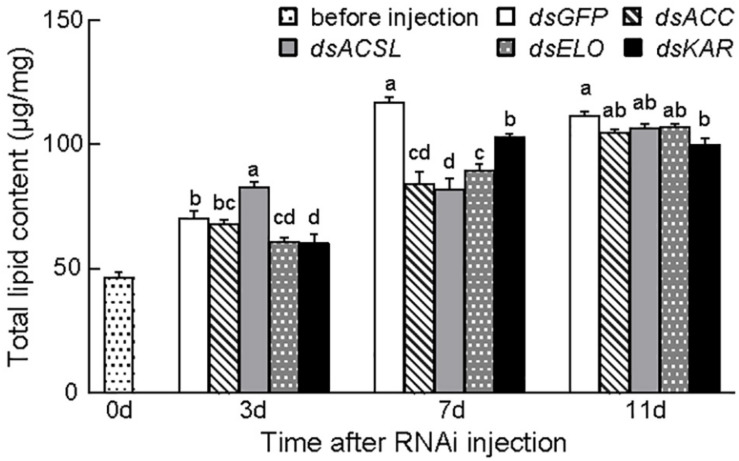
Total lipid levels after dsRNA injection of CsACC, *CsACSL*, *CsELO*, and *CsKAR*. Baseline (before injection) represents the total lipid level content 3 days after eclosion. Data are presented as mean ± SE. Significance analyses were performed using Tukey’s *post-hoc* test. Different letters indicate a significant difference between stages (*P* < 0.05).

The total lipid content in the insects significantly changed 7 days post-injection of dsRNA for gene silencing (*p* < 0.05). Furthermore, the total lipid content (116.95 ± 2.02 μg/mg) significantly decreased and then gradually increased compared with that in the control group (dsGFP). At 11 days after injection, the total lipid content in the dsACC group (104.85 ± 1.33 μg/mg) and dsKAR group (99.76 ± 2.78 μg/mg) remained significantly lower than that in the control group; in the other groups, the difference was no longer significant compared with the control group (Figure 4).

**FIGURE 5:**
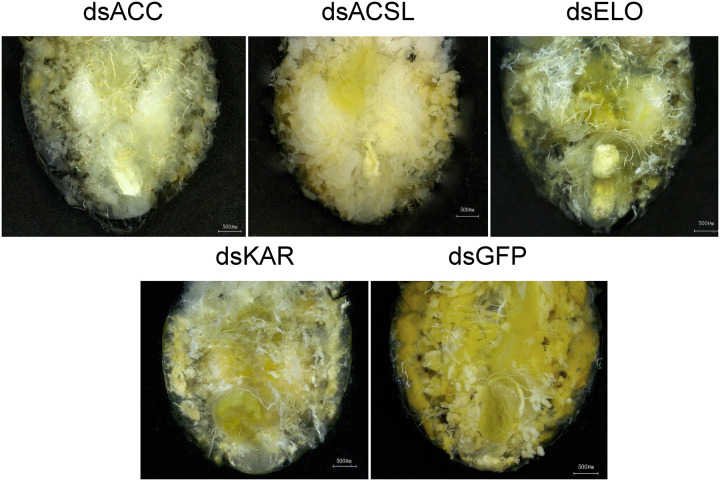
dsACC, dsACSL, dsELO, dsKAR, and dsGFP were injected into the abdomen of a 7-day-old female *Coccinella septempunctata* after rearing for 2 days under diapause-induced conditions.

Morphological differences in *C. septempunctata* were observed 7 days after injection (Figure 5). As shown in the anatomical comparison diagram, the abdominal lipid accumulation in *C. septempunctata* after gene interference was significantly lower than that in the control group (dsGFP), consistent with total lipid content changes.

## Discussion

Diapause regulation has long been a focus of research in the field of biological control; however, understanding the molecular regulation of diapause is still in its infancy (Denlinger, 2002). Although many genes are turned off during diapause, some genes are specifically expressed. The down- or upregulation of various genes or their alterations at the protein level has been reported as a key step for diapause initiation, maintenance, and termination. For example, Güney et al. (2021) identified genes encoding *Leptinotarsa decemlineata* LSD1 and LSD2, and revealed the functions of these proteins in *L. decemlineata* and their roles in diapause and during starvation. In drosophilids, suppressing the expression of Hsp23 andHsp70 by RNAi did not alter the commitment to enter diapause or the duration of diapause, but it is critical for cold survival during diapause in insects (Rinehart et al., 2007). Furthermore, the adipose body-specific expression of two calcium-binding chaperones (LDCRT and LDCNX) in *L. decemlineata* during diapause revealed the same expression pattern in relation to diapause; there was no alteration in females and downregulation in males post-diapause (Doğan et al., 2021).

During the diapause preparation phase, insects store a large amount of lipids, which can be interconverted to sugars and alcohols to provide nutrition and water necessary for biological activities. These lipids serve as the primary energy source for growth during the diapause and post-diapause periods (Arrese and Soulages, 2010). Similarly, adult Colorado potato beetles (*L. decemlineata*) store lipids in fat bodies to meet their energy demands during diapause (De Kort, 1990). In addition, stored lipids are essential for insect resistance to harsh environmental conditions during diapause. We previously found a large amount of fat accumulation in the abdomen of the diapause ladybird, in which the lipid content was two times higher than that in normally developing insects and those in the post-diapause period (Wang et al., 2013).

This study demonstrated that *CsACC*, *CsACSL*, *CsELO*, and *CsKAR* play a pivotal role in lipid accumulation in *C. septempunctata*, as the total lipid content and abdominal lipid accumulation were significantly reduced after gene silencing. We found that the expression of the four genes associated with fatty acid biosynthesis was upregulated during the preparation phase of diapause. As a preliminary speculation, this result suggests that the synthesis of fatty acids is promoted in the preparation phase of diapause to improve lipid accumulation and to prepare for the diapause phase (Hahn and Denlinger, 2007). Consistently, we found that the expression of the four genes decreased in the late diapause and post-diapause periods, approaching the levels observed during the non-diapause period. This may be a mechanism to restore the body composition to normal levels, promoting physiological functions such as mating and oviposition (Ren et al., 2016). Collectively, the results suggest that these four target genes might play an important role in diapause preparation or early diapause.

The changes in the expression profiles of genes throughout the diapause process in *C. septempunctata* are consistent with the findings of a previous study on the enzymes involved in glycometabolism during the diapause process in *Aphidius gifuensis* (Zhang et al., 2020). Here, the temporal expression profile analysis with RNAi further showed that the gene expression significantly decreased 5 and 7 days after injection, indicating a significant interference effect, which could be detected up to 11 days post-injection. Moreover, the lipid content changed with the decrease and recovery of gene expression. According to the anatomical and morphological comparisons, abdominal lipid accumulation after injection significantly reduced compared with that in the control group (dsGFP), consistent with the measured changes in the total lipid content. This further confirmed that the four target genes can regulate lipid accumulation in insects and play an important role in the fatty acid synthesis pathway. Reynolds et al. (2012) assessed the relative mRNA abundance of 21 genes and detected differential expression of 10 genes in *Aedes albopictus*. Ten genes were differentially expressed in a manner that suggests they have a role in diapause and that the abundance of transcriptional genes regulating lipid storage (lsd2), lipolysis (lip2, lip3, and lip4), β-oxidation (acs, cpt, acd4, and acd5), and unsaturated fatty acid synthesis (desat and face) significantly changed. These data suggest that transcriptional changes in multiple processes contribute to an increase in the amount of lipids in diapause embryos. Tan et al. (2017) have shown that one of the proteins upregulated in the head of diapause-committed *Colaphellus bowringi* females, that is, FABP, is critical for fat accumulation during diapause preparation. During the preparation for diapause, the lipid content increases, and the ratio of fatty acid content to unsaturated fatty acids also affects the cold tolerance of insects, which can increase solute concentrations and slow metabolism. This mechanism might also be an adaptation to withstand adverse environments, which is conducive to survival during diapause (Danks, 2000).

Other key genes involved in fatty acid metabolism have also been reported. Toprak et al. (2014) identified two LSD (EmLSD1-2) orthologs in the hemimetabolous sunn pest (*Eurygaster maura*), expressed continuously throughout the life cycle of the insect, but peak in the fourth nymphal stage. Furthermore, a good coordination between perilipin gene expression and the physiological and biological activities was observed in the pest. The dynamic coordination of gene expression may be a key process to regulate lipid metabolism in sunn pest. Saraiva et al. (2021) investigated the regulation of *Rhodnius prolixus* ACC (RhoprACC) expression and *de novo* lipogenesis activity in adult females under different nutritional and metabolic conditions. In the fat body, gene expression increased in fasted females, and it decreased after a blood meal. This downregulation in RhoprACC occurred despite an increase in fat body *de novo* lipogenesis after a blood meal, suggesting the primary regulator of feeding-induced lipogenesis might vary between species. In this study, diapause was initiated and the expression of the *CSACC* gene was upregulated, consistent with the upregulation of fast *R. prolixus* female RhoprACC. However, the upregulation of *CsACC* in *C. septempunctata* promotes the synthesis of fatty acids throughout the diapause stage. For example, Sim and Denlinger (2009) identified the regulatory genes of fat storage and consumption during diapause in *Culex pipiens* and found that fatty acid synthetase (*FAS*)-1 gene expression was upregulated during early diapause. After RNAi to silence *FAS1* and *FAS3*, female mosquitoes failed to sequester the lipids required for overwintering, demonstrating that these two fatty acids play an essential role in fat storage during early diapause. In addition, reduced *FAS1* expression has been reported to hinder diapause in *Colaphellus bowringi* Baly, and *FAS2* interference reduced lipid storage, affecting the expression of stress tolerance-related genes and increasing the body water content (Liu et al., 2016). Moreover, *ACC* and *FAS1* knockout in *Aedes aegypti* reduced lipid biosynthesis rate in adipose bodies and midgut digestion (Alabaster et al., 2011). These results matched the expression trend of diapause-associated genes in lipid metabolism, which resulted in decreased lipid storage in *C. septempunctata*. Identification of the basic regulatory factors of lipid metabolism in *Drosophila* can provide a new direction for controlling metabolic pathways (Baker and Thummel, 2007). Similarly, the four target genes that play an important role in regulating the fatty acid metabolism pathway in *C. septempunctata* can provide a theoretical basis for revealing the molecular mechanism of diapause, which can be used in biological control strategies.

To further elucidate the molecular mechanism underlying the diapause process in *C. septempunctata*, the relationship between the lipid metabolism pathway and upstream and downstream pathways should be determined in the subsequent studies. Furthermore, key genes regulating the diapause process, should be identified, which will provide new guidance for biological control with *C. septempunctata*.

## Conclusion

In this study, the functions of key enzymes involved in fatty acid synthesis in *C. septempunctata* were investigated; the full-length genes of *CsACC*, *CsACSL*, *CsELO*, and *CsKAR* were cloned; and their transcriptional levels were investigated under different stages—diapause induction, non-diapause, and post-diapause. The four fatty acid-related genes were upregulated during the preparation for diapause, and their downregulation with RNAi reduced the lipid content. These results demonstrate that these four enzymes could promote fat synthesis and accumulation, and affect the diapause process. The results could help explore the gene regulatory network of fat metabolism in the diapause process and, more broadly, provide new research directions into diapause regulation. Although preliminary, these findings warrant further research to identify more genes associated with metabolic pathways for exploring the molecular mechanisms of diapause and lipid accumulation.

## Data Availability Statement

The datasets presented in this study can be found in online repositories. The names of the repository/repositories and accession number(s) can be found in the article/Supplementary Material.

## Author Contributions

MX and L-SZh designed the research. MX performed the experiments, analyzed the data, and wrote the manuscript. H-ZZ, X-YJ, M-QW, J-JM, and Y-YL provided the technical assistance. L-SZh and L-SZa revised the manuscript. All authors contributed to the article and approved the submitted version.

## Conflict of Interest

The authors declare that the research was conducted in the absence of any commercial or financial relationships that could be construed as a potential conflict of interest.

## Publisher’s Note

All claims expressed in this article are solely those of the authors and do not necessarily represent those of their affiliated organizations, or those of the publisher, the editors and the reviewers. Any product that may be evaluated in this article, or claim that may be made by its manufacturer, is not guaranteed or endorsed by the publisher.

## Footnotes

1
https://www.ncbi.nlm.nih.gov/orffinder/

2
http://web.expasy.org/protparam/

3
http://www.cbs.dtu.dk/services/TMHMM/

4
https://www.ncbi.nlm.nih.gov/Structure/cdd/wrpsb.cgi

5
https://blast.ncbi.nlm.nih.gov/Blast.cgi

6
https://www.ncbi.nlm.nih.gov/genbank/
