# Supplementary material for: Sequencing, Expression, and Functional Analyses of Four Genes Related to Fatty Acid Biosynthesis During the Diapause Process in the Female Ladybird, Coccinella septempunctata L
Source: Front Physiol. 2021 Aug 19;12:706032. doi: 10.3389/fphys.2021.706032 (PMC8417001; doi:10.3389/fphys.2021.706032)
Supplement: Supplementary file 1 [file Image_1.pdf]

Supplementary Material

CsACC

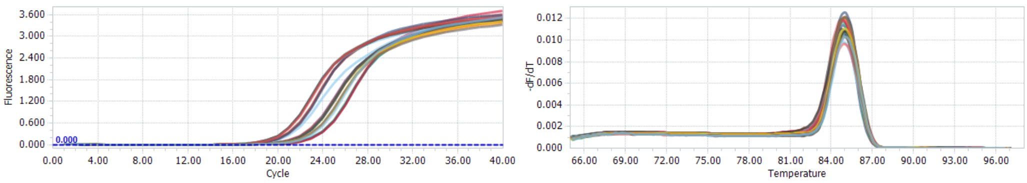

CsACSL

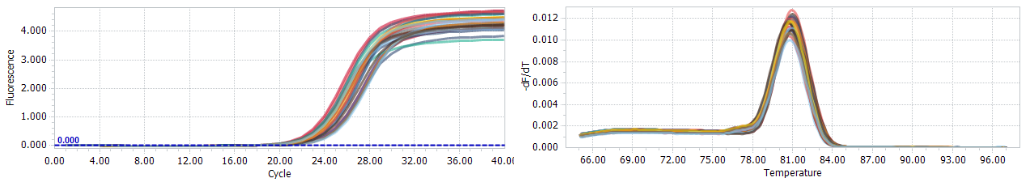

CsELO

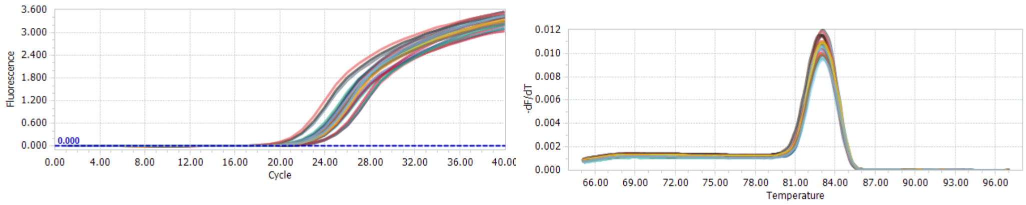

CsKAR

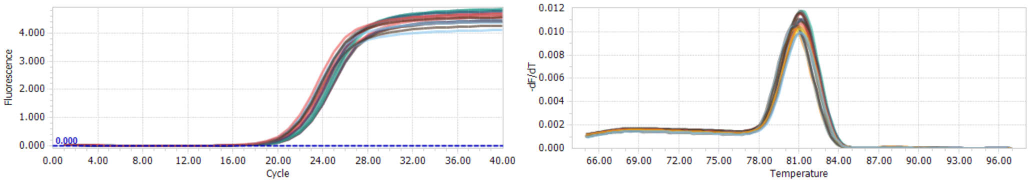

Actin

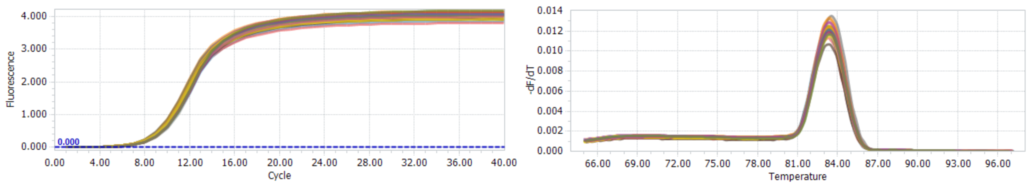

Supplementary Figure 1. Amplification plot and dissociation curve of CsACC, CsACSL, CsELO, CsKAR, and actin
