# Supplementary material for: Sequencing, Expression, and Functional Analyses of Four Genes Related to Fatty Acid Biosynthesis During the Diapause Process in the Female Ladybird, Coccinella septempunctata L
Source: Front Physiol. 2021 Aug 19;12:706032. doi: 10.3389/fphys.2021.706032 (PMC8417001; doi:10.3389/fphys.2021.706032)
Supplement: Supplementary file 2 [file Image_2.pdf]

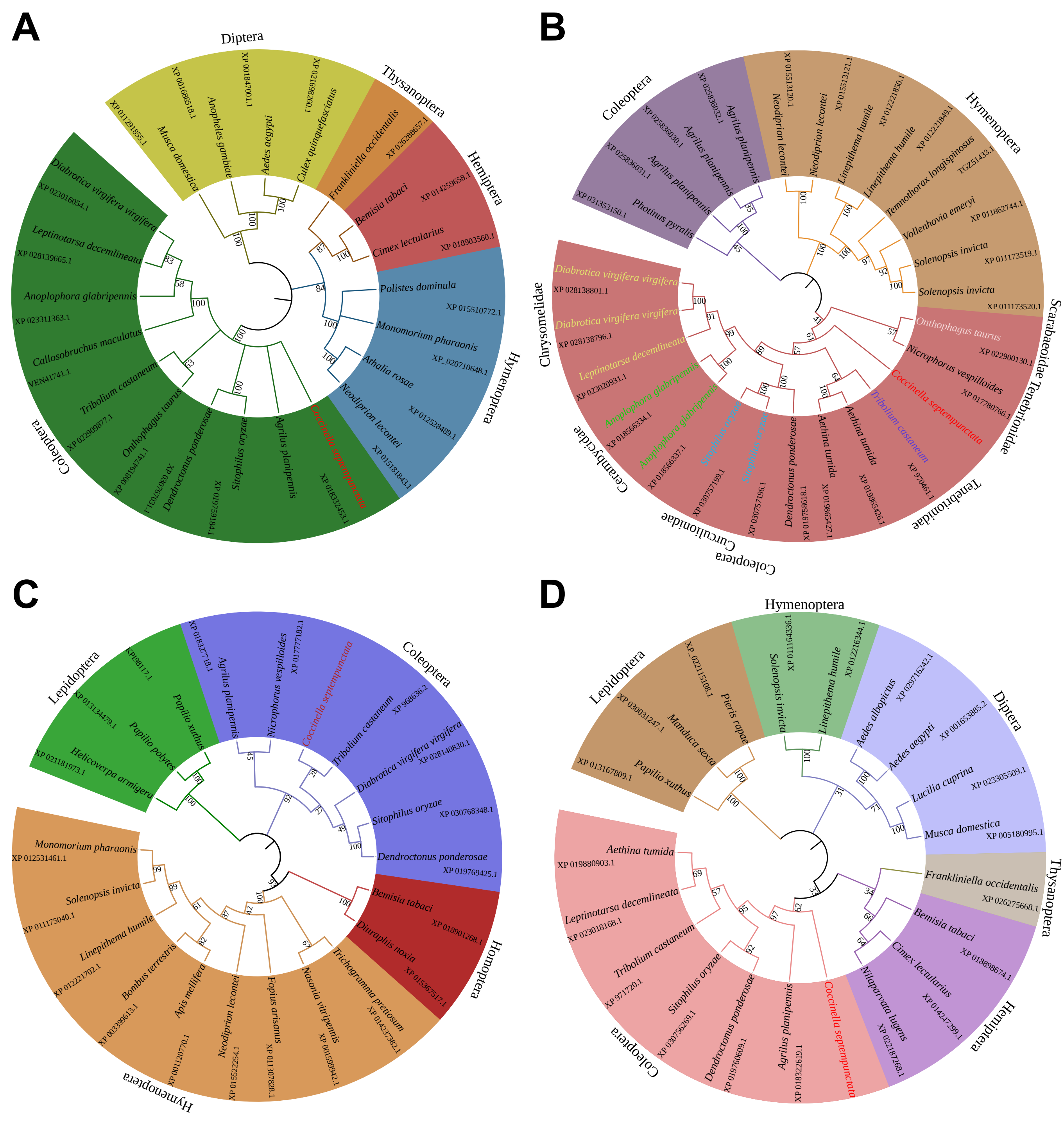
A
Diptera
Thysanoptera
Hemiptera
Hymenoptera
Coleoptera
B
Coleoptera
Hymenoptera
Scarabaeoidae
Tenebrionidae
Tenebrionidae
Chrysomelidae
Cerambycidae
Curculionidae
Coleoptera
C
Lepidoptera
Coleoptera
Homoptera
Hymenoptera
D
Hymenoptera
Lepidoptera
Diptera
Thysanoptera
Hemiptera
Coleoptera
